# Near-distance raw and reconstructed ground based SAR data

**DOI:** 10.1016/j.dib.2023.109620

**Published:** 2023-09-25

**Authors:** Filip Turčinović, Marin Kačan, Dario Bojanjac, Marko Bosiljevac

**Affiliations:** University of Zagreb, Faculty of Electrical Engineering and Computing, Unska 3, 10 000 Zagreb, Croatia

**Keywords:** Synthetic aperture radar, Ground based SAR, Raw radar data, Radar image, Object classification

## Abstract

Presented data includes two datasets named RealSAR-RAW and RealSAR-IMG. The first one contains unprocessed (raw) radar data obtained using Ground Based Synthetic Aperture Radar (GBSAR), while the second one contains images reconstructed using Omega-K algorithm applied to raw data from the first set. The GBSAR system moves the radar sensor along the track to virtually extend (synthesize) the antenna aperture and provides imaging data of the area in front of the system. The used sensor was a Frequency Modulated Continuous Wave (FMCW) radar with a central frequency of 24 GHz and a 700 MHz wide bandwidth which in our case covered the observed scene in 30 steps with 1 cm step size. The measured (recorded) scenes were made on combinations of three test objects (bottles) made of different material (aluminum, glass, and plastic) in different positions. The aim was to develop a small dataset of GBSAR data useful for classification applications focused on distinguishing different materials from sparse radar data.

Specifications Table

**Table:** 

Subject	Computer Science
Specific subject area	Signal Processing
Type of data	Text Image
How the data were acquired	Raw data was obtained using Ground Based SAR system developed by our laboratory group. The system is based on Raspberry Pi microcomputer and 24 GHz FMCW module. The images were reconstructed using Omega-K algorithm applied on raw radar data.
Data format	Raw and reconstructed
Description of data collection	The recordings of near distanced objects were conducted using GBSAR in stop-and-go mode with 30 cm total aperture and step size set to 1 cm. The matrix from each measurement is stored to RealSAR-RAW in .txt format, while its image pandan to RealSAR-IMG in .png format. All measurements were performed in real-world environment (non-ideal with possible minor reflections from background objects).
Data source location	· Institution: University of Zagreb, Faculty of Electrical Engineering and Computing · City/Town/Region: Zagreb · Country: Croatia · Latitude and longitude (and GPS coordinates, if possible) for collected samples/data: 45.8014974,15.9707206
Data accessibility	Repository name: Mendeley data Data identification number: 10.17632/m458grc688.2 Direct URL to data: 10.3390/rs14225673
Related research article	Kačan M, Turčinović F, Bojanjac D, Bosiljevac M. Deep Learning Approach for Object Classification on Raw and Reconstructed GBSAR Data. Remote Sensing. 2022; 14(22):5673. 10.3390/rs14225673

## 1 Value of the Data

• Raw radar data is a collection of signals recorded for several near-distanced objects and stored in the form of a matrix. These signals were recorded using custom Ground Based SAR described later and based on the provided parameters researchers can use these data to develop and test various radar image reconstruction algorithms.
• Each line of the raw data matrix represents a single FMCW radar signal. These signals can be independently extracted and used to test and study various signal processing techniques that can facilitate different image reconstructions or other data representations.
• The provided dataset also contains reconstructed radar images obtained from the given raw radar data. These images were obtained using Omega-K [2] reconstruction algorithm and they can serve as benchmarks for evaluating different implementations of the same algorithm or for comparison with images reconstructed using different algorithms.
• Both datasets, raw radar data and reconstructed images can be used to develop and evaluate models and systems for automated object classification.
• Image reconstruction algorithms in their process introduce certain approximations when handling the data. By comparing the object classifications based on raw and reconstructed datasets researchers can detect the level of influence certain approximations or certain algorithms have on the integrity of the data.

## 2 Objective

The dataset contains raw radar data recorded using Ground Based Synthetic Aperture Radar (GBSAR) in a realistic environment and images reconstructed using that data. This dataset provides the measurements of the scenes in which objects are set in near distance (in 1 m range from the radar). The measurements in raw format may be used to test various reconstruction algorithms and also to manipulate the data on signal level. Such format can also be utilized in raw-data based object classification, while reconstructed images in image-data based object classification. In [1] the comparison between those two approaches based on ResNet18 architecture is given. However, various deep learning models based on both raw and reconstructed radar data can be implemented using this dataset.

## 3 Data Description

**Fig. 1:**
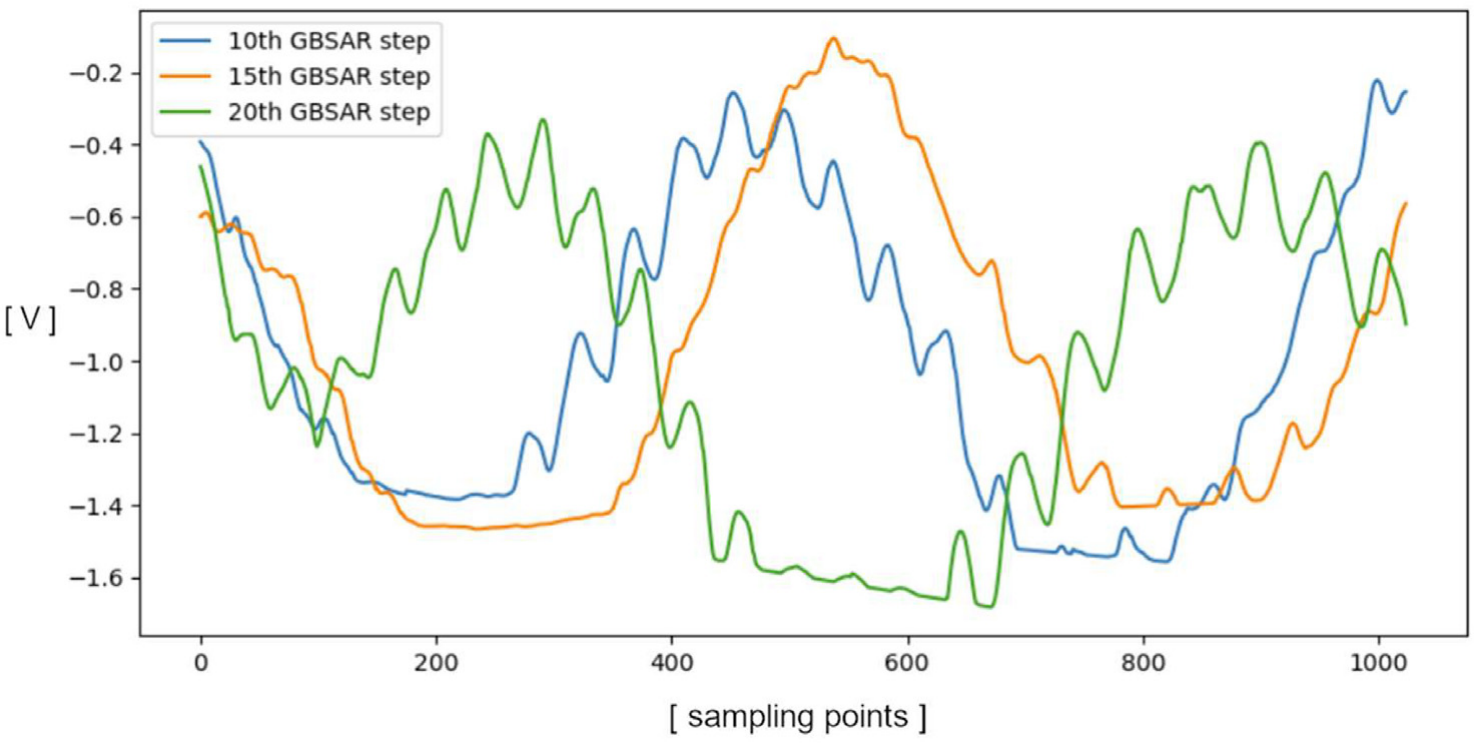
FMCW signals in 10th, 15th and 20th GBSAR step.

The repository is divided into two parts: RealSAR-RAW and RealSAR-IMG. The first one contains raw radar data obtained using Ground Based SAR in form of matrix (in .txt format). In the conducted measurements, GBSAR moved Frequency Modulated Continuous Wave (FMCW) sensor for 30 steps and in each of them stored the resulting voltage signal after mixing represented with 1024 points as one column of the output matrix. Therefore, the output matrix from the GBSAR system has the dimensions 1024 × 30. Three example FMCW signals from three steps (10th, 15th and 20th) of a scene with aluminum bottle (0_Aluminum.txt) are shown in Fig. 1.

The radar image is reconstructed based on that matrix. After processing the data with the Omega-K [2] reconstruction algorithm, the radar image presented in the form of heatmap is stored in .png format (496 × 369 px).

**Fig. 2:**
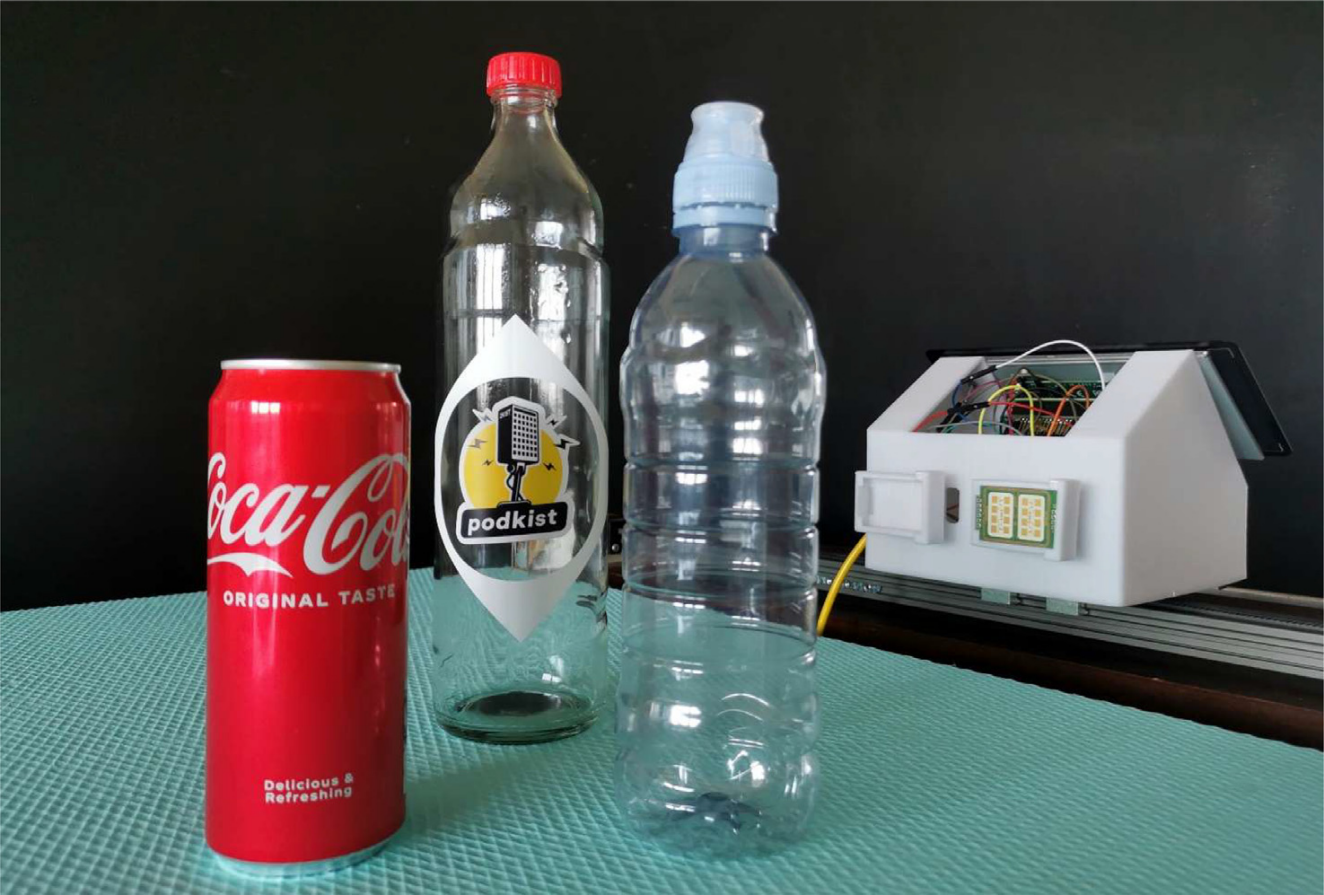
Test objects and GBSAR-Pi system [1].

As mentioned, the observed scenes include various position combinations of three test objects: aluminum, glass and plastic bottle. In a total of 337 scenes, 172 of them had an aluminum bottle, 172 had glass bottle, while 179 of them had a plastic bottle. 29 scenes were recorded without objects. Test objects and GBSAR-Pi used in the measurements are shown in Fig. 2.

The name of each example is the same in both RAW and IMG sets. It consists of id of the example, (capitalized) names of bottles present in that scene, and filename extension in this order:

{id_of_the_example}_{Names_of_bottles}.{filename_extension}.

**Fig. 3:**
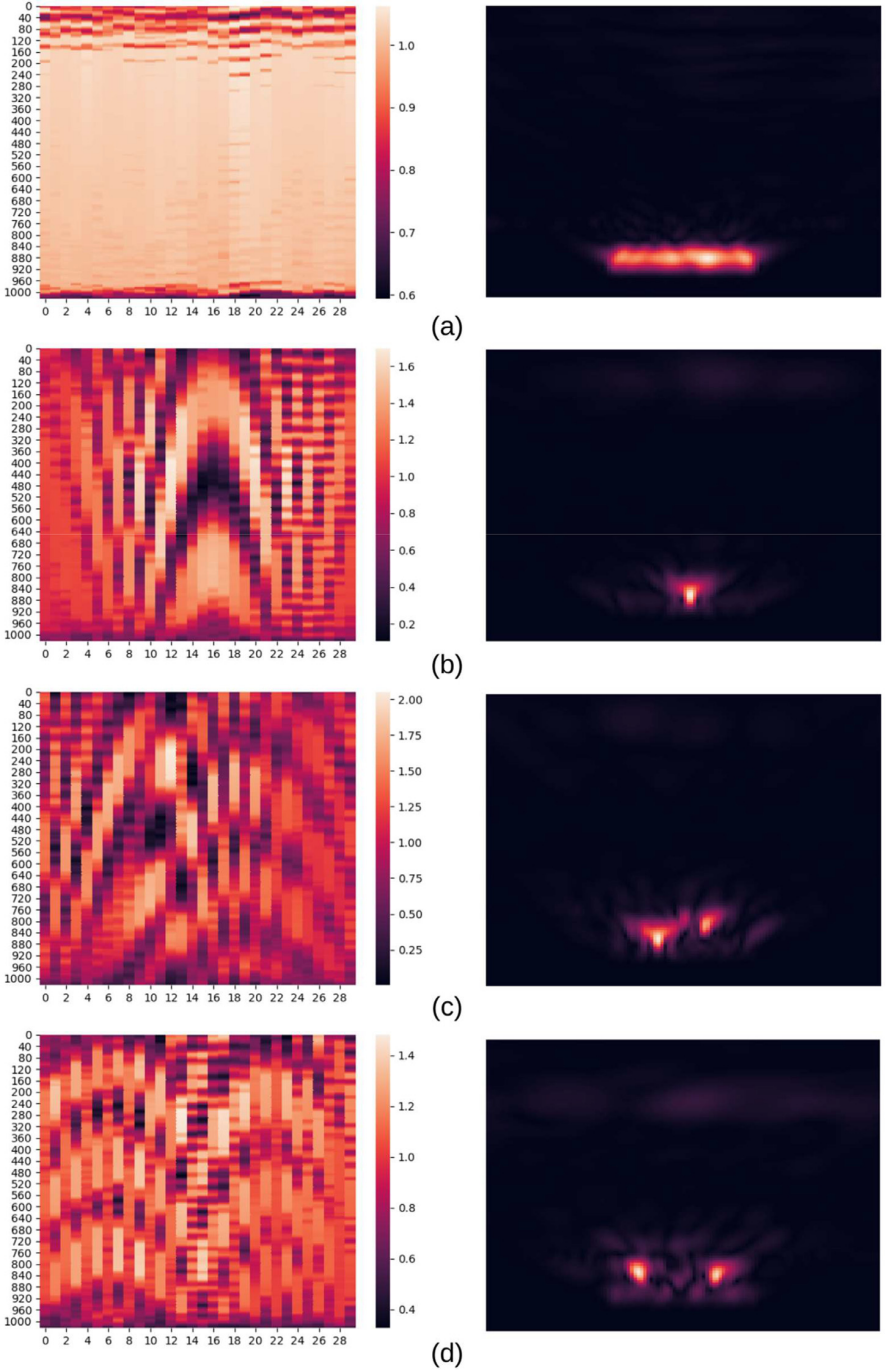
Pairs of RealSAR-RAW (left) and RealSAR-IMG (right) examples. Example pair (a) represents an empty scene, (b) scene with an aluminum bottle, (c) scene with an aluminum and a glass bottle, while (d) contains all three bottles [1].

For example, if the scene included aluminum and glass bottles, and had id 94, its filename in RealSAR-RAW set is 94_AluminumGlass.txt, and in RealSAR-IMG 94_AluminumGlass.png. It is important to emphasize that id of the example is the connection between two sets. Hence, 94_AluminumGlass.png is reconstructed using 94_AluminumGlass.txt. Examples of raw and reconstructed data is shown in Fig. 3. Specifically, an example of a scene with aluminum and glass bottles is shown in Fig. 3(c).

## 4 Experimental Design, Materials and Methods

### 4.1 GBSAR and Omega-K

Ground Based Synthetic Aperture Radar (GBSAR) is a type of remote sensing imaging system that uses the motion of the sensor to virtually extend its antenna. Unlike typical airborne SAR, GBSAR emits radiation perpendicular to its moving path, covering the area in front of it. The sensor provides distance information for each position along its path by using linear FMCW (Frequency Modulated Continuous Wave) radar signals, which are called chirps meaning that their frequency is changed linearly during transmission. The chirps reflect from observed objects with the same frequency change and arrive with a delay to the receiver, and when mixed with emitted chirps, the resulting signal will have a frequency that is proportional to the distance between the radar and the object.

**Fig. 4:**
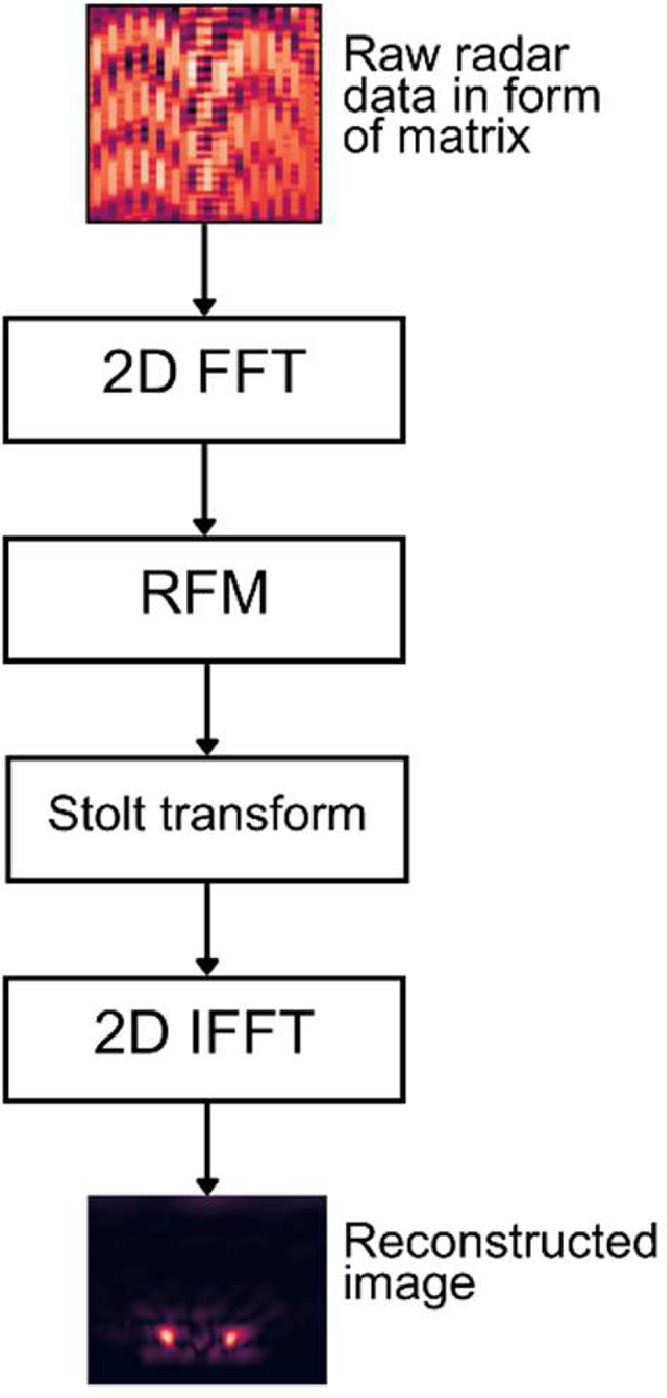
Omega-K reconstruction algorithm steps.

Different sensor positions generate a set of such signals, which can be used to create a two-dimensional radar image of the observed area using image reconstruction algorithms, such as Omega-K [2]. The Omega-K algorithm processes raw data in the frequency domain to filter out unwanted signal components and interpolate unevenly spaced data points, after which an image in range-azimuth space is generated using inverse Fourier transformation in both axes. The block diagram of Omega- K algorithm is shown in Fig. 4 depicting the major steps. Raw data is first transformed into frequency domain using two-dimensional Fourier transformation (commonly implemented using Fast Fourier Transform algorithm, 2D FFT) after which a step called Reference Function Multiplication (RFM) is performed. This step corresponds to a process commonly known as matched filtering in digital communication theory and ensures optimal signal-to-noise ratio of the signal. This step is followed by Stolt transformation which modifies the geometry of the problem in order to correspond to linear x-y coordinates. This correspondingly allows the use of two-dimensional inverse Fourier transformation (2D IFFT) in the final step which results in the reconstructed radar image in x-y coordinate space. Exact details of the algorithm and the mathematical implementation can be found in [2] and our algorithm implementation used in this work is available in [4].

### 4.2 Implementation

**Fig. 5:**
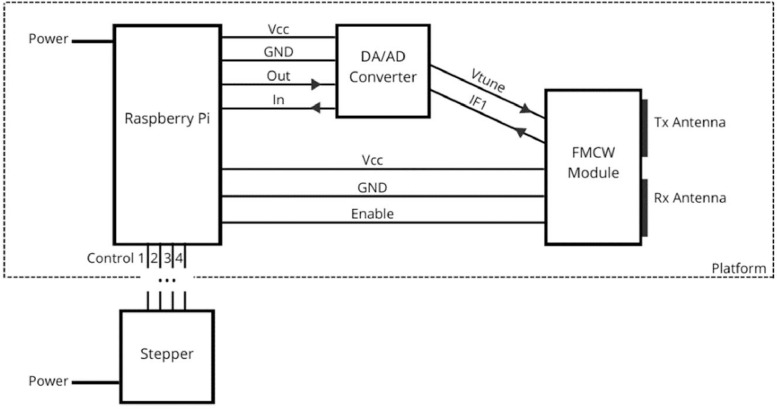
GBSAR-Pi scheme.

To generate a database, GBSAR-Pi was developed. GBSAR-Pi is a GBSAR system based on the Raspberry Pi microcomputer and Innosent IVS-362 FMCW radar module [3], and the complete scheme is given in Fig. 5.

The used FMCW module (IVS-362) operates in the 24 GHz band and includes integrated transmitting and receiving antennas as shown in the scheme, and it also includes a mixer and a voltage-controlled oscillator. For operation the used FMCW module requires DC power (*V_cc_* = 5 V) and “*Enable*” signal (voltage High) to activate the module. Most importantly, the *V_tune_* input of the module is the input for the chirp voltage signal which controls the output frequency sweep of the module thus enabling the FMCW operation. The output of the module is *IF1* and it represents the output of the mixer where the reference signal and signal reflected from the object are combined. Signal IF1 is the main FMCW output signal and its frequency corresponds to the distance of the objects in front of the radar module. This signal is passed through AD converter and it represents the main input signal to RPi and it is the basis of the raw dataset presented here (individual FMCW signals from our dataset are depicted in Fig. 1).

The entire GBSAR platform, which contains the Raspberry Pi, AD/DA converter, FMCW module, and a signal amplifier, is custom 3D-printed for precise linear movement and polarization change (white GBSAR-Pi platform is shown in Fig. 2). The system operates in stop-and-go mode, emitting multiple signals in each step and storing the average of received ones to maximize the signal-to-noise ratio (SNR). The obtained matrix of average signals from each step is stored locally (on RPi) and sent to the server. The FMCW signals transmitted by the module have a central frequency of 24 GHz and a sweep range of 700 MHz bandwidth, which provides a range resolution of the system of 21.4 cm. Each sweep in the emitted signal is generated by the RPi with 1024 frequency points and has a duration of 166 ms, giving a chirp rate change of frequency of 4.2 × 10^9. That signal is marked as “Out” from RPi and it is then passed through DA converter to control the FMCW module as *V_tune_*. The number of steps and step size are adjustable with a maximum aperture of 1 m. The system is fully autonomous and controlled by a display set on the platform and an optional external keyboard.

The image reconstruction algorithm Omega-K was implemented using the Python programming language and additional libraries, including numpy, scipy, and seaborn which provide the necessary functions for FFT, IFFT, interpolation, and visualization. The program code is adjustable for central frequency, bandwidth, chirp duration and step size, and is fully given in [4].

### 4.3 Measurements

The measurements were intentionally conducted in a cluttered room to introduce additional noise. Test objects were empty bottles of similar size and shape, made of different materials (aluminum, glass, and plastic). Hence, they can be distinguished based on their reflectance. Any of eight possible subsets of three objects (including an empty scene without any objects) could appear in a given scene. Additionally, different object positions in azimuth and range directions, as well as varying polarizations used for recording, further added complexity to the scene. It's worth noting that a scene could have at most one object of a certain material. The step size was set to 1 cm, while the total aperture to 30 cm. All GBSAR parameters used in the measurements are given in Table 1.

**Table 1:** Scene and GBSAR parameters

# of scene combinations	8
# of measurements	338
Step size	1 cm
Azimuth points	30
Total aperture length	30 cm
Polarization	HH and VV
FMCW signal frequency	24 GHz
Bandwidth	700 MHz
Chirp duration	166 ms
# of frequency points	1024

## 5 Limitations

The presented dataset was recorded in a realistic scenario with possible interfering objects in the background of the objects of interest. This was intentional to ensure that we obtain data that can be useful in deep learning classification applications and although this dataset is limited in size it presents a very useful tool for studying SAR algorithms, implementations and related classification methods.

## Ethics Statement

The authors have read and follow the ethical requirements for publication in Data in Brief. The current work does not involve human subjects, animal experiments, or any data collected from social media platforms.

## CRediT authorship contribution statement

**Filip Turčinović:** Conceptualization, Methodology, Software, Validation, Formal analysis, Investigation, Data curation, Writing – original draft, Visualization. **Marin Kačan:** Conceptualization, Methodology, Validation, Formal analysis, Investigation, Writing – review & editing, Supervision. **Dario Bojanjac:** Conceptualization, Methodology, Validation, Writing – review & editing, Supervision. **Marko Bosiljevac:** Conceptualization, Methodology, Validation, Resources, Writing – review & editing, Supervision, Project administration, Funding acquisition.

## Data Availability

Near-Distance Raw and Reconstructed Ground Based SAR Data (Original data) (Mendeley Data)
